# Targeted infection of HIV-1 Env expressing cells by HIV(CD4/CXCR4) vectors reveals a potential new rationale for HIV-1 mediated down-modulation of CD4

**DOI:** 10.1186/1742-4690-2-80

**Published:** 2005-12-21

**Authors:** Zhiping Ye, George G Harmison, Jack A Ragheb, Manfred Schubert

**Affiliations:** 1 Molecular Virology and Neurogenetics Section, National Institute of Neurological Disorders and Stroke, National Institutes of Health, Rm. 4S-18, 5625 Fishers Lane, Bethesda, MD 20892-9403, USA; 2 Laboratory of Pediatric and Respiratory Viral Diseases, Center for Biologics Evaluation and Research, Food and Drug Administration, Bldg. 29A, 8800 Rockville Pike, Bethesda, MD 20892, USA; 3 Neurogenetics Branch, National Institute of Neurological Disorders and Stroke, National Institutes of Health, Bldg. 35, Rm. 2A1012, 35 Convent Drive, Bethesda, MD 20892-3705, USA; 4 Clinical and Molecular Immunology Section, National Eye Institute, National Institutes of Health, Bldg. 10, Rm. 10N113A, 10 Center Drive, Bethesda, MD 20892-1857, USA

## Abstract

**Background:**

Efficient targeted gene transfer and cell type specific transgene expression are important for the safe and effective expression of transgenes *in vivo*. Enveloped viral vectors allow insertion of exogenous membrane proteins into their envelopes, which could potentially aid in the targeted transduction of specific cell types. Our goal was to specifically target cells that express the T cell tropic HIV-1 envelope protein (Env) using the highly specific interaction of Env with its cellular receptor (CD4) inserted into the envelope of an HIV-1-based viral vector.

**Results:**

To generate HIV-1-based vectors carrying the CD4 molecule in their envelope, the CD4 ectodomain was fused to diverse membrane anchors and inserted together with the HIV-1 coreceptor CXCR4 into the envelopes of HIV-1 vector particles. Independent of the type of CD4 anchor, all chimeric CD4 proteins inserted into HIV-1 vector envelopes and the resultant HIV(CD4/CXCR4) particles were able to selectively confer neomycin resistance to cells expressing the fusogenic T cell tropic HIV-1 Env protein. Unexpectedly, in the absence of Env on the target cells, all vector particles carrying the CD4 ectodomain anchored in their envelope adhered to various cell types without infecting these cells. This cell adhesion was very avid. It was independent of the presence of Env on the target cell, the type of CD4 anchor or the presence of CXCR4 on the particle. In mixed cell populations with defined ratios of Env^+^/Env^- ^cells, the targeted transduction of Env^+ ^cells by HIV(CD4/CXCR4) particles was diminished in proportion to the number of Env^- ^cells.

**Conclusion:**

Vector diversion caused by a strong, non-selective cell binding of CD4^+^-vector particles effectively prevents the targeted transduction of HIV-1 Env expressing cells in mixed cell populations. This Env-independent cell adhesion severely limits the effective use of targeted HIV(CD4/CXCR4) vectors designed to interfere with HIV-1 replication *in vivo*. Importantly, the existence of this newly described and remarkably strong CD4-dependent cell adhesion suggests that the multiple viral efforts to reduce CD4 cell surface expression may, in part, be to prevent cell adhesion to non-target cells and thereby to increase the infectivity of viral progeny. Preventing CD4 down-modulation by HIV-1 might be an effective component of a multi-faceted antiviral strategy.

## Background

The interaction of the human immunodeficiency virus type 1 (HIV-1) envelope protein (Env) with its cellular receptor CD4 [1] was recognized early as an opportunity to selectively inhibit HIV-1 infection by competition with soluble CD4 or by targeting HIV-1 infected cells with cytocidal molecular conjugates of CD4 such as CD4-Pseudomonas exotoxin [2,3]. CD4 is a transmembrane protein and can be inserted into viral envelopes [4,5]. We postulated that defective, CD4 encoding HIV-1 vectors could be designed to target HIV-1 infected cells and to interfere with HIV-1 replication. In a role reversal, such HIV(CD4) particles would target HIV-1 Env^+ ^cells with Env providing membrane fusion activity. Defective HIV-1 genomes that interfere with HIV-1 replication by expression of a chimeric CD4 protein and/or a multitarget-ribozyme were previously designed in our lab [6,7]. Several replication competent enveloped viral vectors that are able to target HIV-1 infected cells were developed from vesicular stomatitis virus (VSV) [8] and rabies virus [9]. Targeted retroviral vectors have previously been used including avian leucosis virus (ALV) [4], Moloney murine leukemia virus [10], HIV-1 and simian immunodeficiency virus type 1 [11]. Most of these particles carry, in addition to CD4, one of the coreceptors CXCR4 or CCR5 in their envelopes.

Altering viral envelopes to target select cell types remains a challenge and mechanisms for the sorting and insertion of membrane proteins into viral envelopes are not fully understood [12]. Some enveloped viruses like rabies virus appear more restrictive with respect to the origin of the cytoplasmic domain for insertion of CD4 and CXCR4 [9] as compared to VSV [13], a related rhabdovirus. HIV-1 appears less restrictive with respect to insertion of foreign membrane proteins [13-18]. Extensive studies identified many cellular membrane proteins, which are inserted into the envelopes of budding viruses (for review see [19]). Insertion of cellular ICAM-1, for example, can promote HIV-1 infection of specific cell types such as CD4^+ ^T-lymphocytes and memory CD4^+ ^T cells [20-22] and will probably influence viral propagation *in vivo*.

In this communication, we focus on the functional activities of several chimeric CD4 proteins after their insertion into HIV-1 envelopes. During wild-type HIV-1 infection, insertion of CD4 into HIV-1 envelopes is efficiently blocked by mechanisms that involve the viral Vpu, Nef and Env proteins [23,24]. As a result, CD4 down-modulation increases Env expression at the cell membrane for better insertion into viral envelopes and thereby increases virus release and infectivity [25-32] while preventing superinfection of the HIV-1 infected cells.

An inverse relationship between the amount of surface CD4 expression and the infectivity and release of viral progeny has been described [33]. Previous studies on the effect of CD4 on HIV-1 infectivity and particle release were carried out when some of the viral mechanisms for CD4 down-modulation were still active. The present study describes the insertion of diverse chimeric CD4 proteins into HIV-1 vector envelopes in the absence CD4 down-modulation. This communication focuses on the consequences of inserting CD4 into HIV-1 vector envelopes. The study reports a novel, highly effective CD4-dependent cell binding activity, which does not reduce the specificity of targeting cells expressing T cell tropic HIV-1 Env protein but greatly reduces its efficacy in heterogeneous cell populations. These findings may provide a new rationale for the virally mediated down-modulation of CD4, which could have important implications for HIV-1 infectivity *in vivo*.

## Results

### Structure and expression of chimeric CD4 proteins

**Figure 1:**
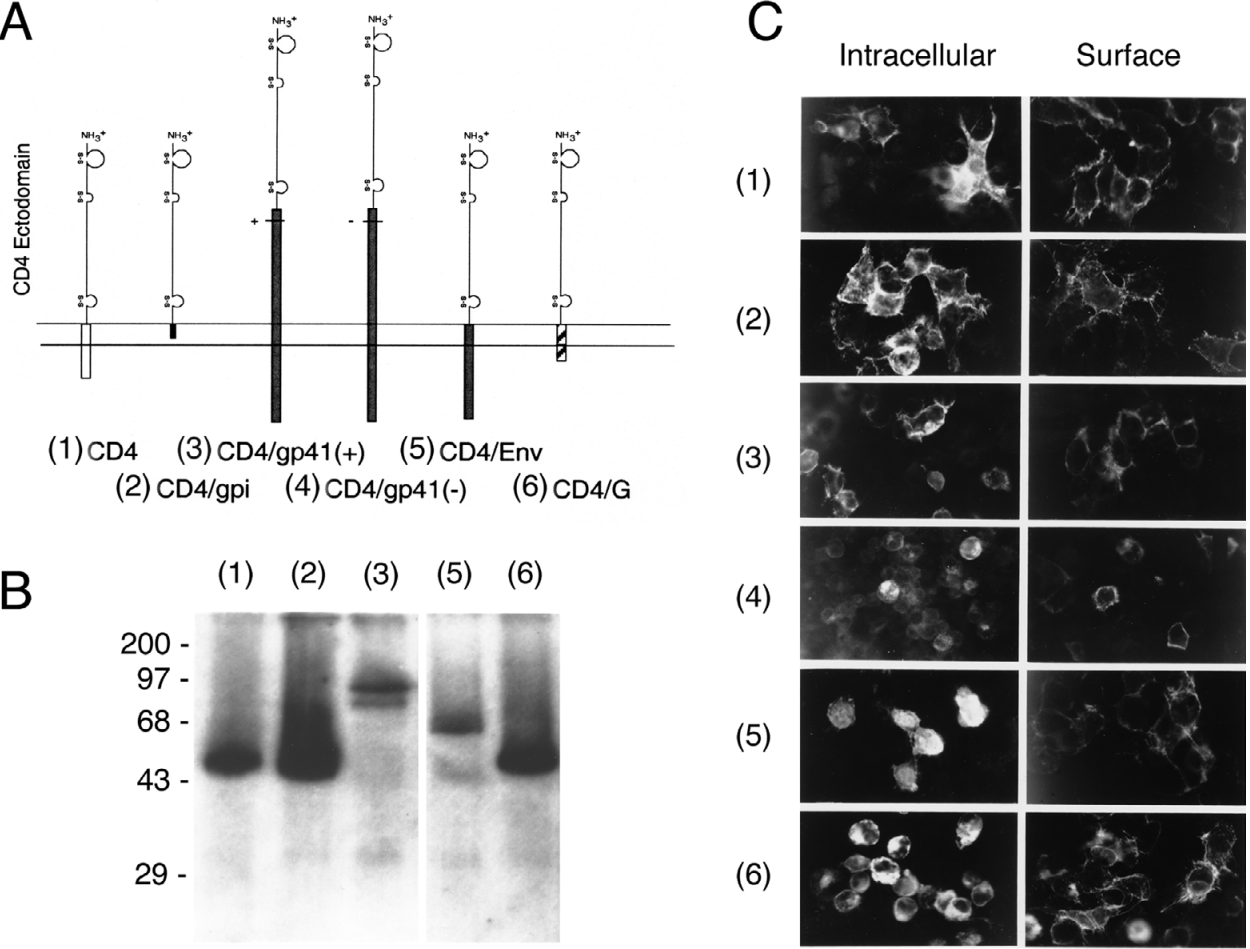
**Chimeric CD4 proteins. **(A) Membrane anchors. The CD4 ectodomain of CD4 (1) was linked to five heterologous membrane anchors: the glycosylphosphatidyl inositol region of the DAF protein (2), the transmembrane and cytoplasmic domains of either HIV-1 Env (5) or the VSV G (6), or the entire gp41 region of HIV-1 Env with (3) or without (4) the proteolytic cleavage site. (B) Western blot analysis of CD4 and the chimeric CD4 proteins. The six proteins were expressed from the CMV promoter in pCR3 after transfection of Hek293 cells and analyzed by Western blot using anti-CD4 antibodies. (C) Intracellular and cell surface expression. Intracellular expression of chimeric CD4 proteins after DNA transfection in HeLa cells was evaluated using immunofluorescent staining of fixed cells after cell permeabilization and compared to cell surface expression of CD4 without cell permeabilization.

For the targeting of cells that express HIV-1 Env, several chimeric CD4 proteins were assembled by PCR DNA fusion (Figure 1A). Each protein contained 397 amino acids of the CD4 ectodomain including the signal peptide. To compare the potential roles of the CD4 membrane anchor and cytoplasmic domains during vector assembly, the CD4 ectodomain was fused to a glycosylphosphatidylinositol (gpi) anchor [34], or it was fused to the transmembrane and cytoplasmic tail regions of either VSV G protein or the HIV-1 Env protein. Two additional CD4 constructs contained the CD4 ectodomain fused to the entire gp41 region of HIV-1 Env. The amino end of the gp41 region was extended into the gp120 region of Env, 20 amino acids beyond the proteolytic cleavage site at the gp120/gp41 junction. The Ile-Glu cleavage site itself was either left unchanged (CD4/gp41^+^) or it was deleted (CD4/gp41^-^).

Native CD4 and the five chimeric CD4 proteins were expressed after DNA transfection of Hek293 cells. The proteins migrated according to their predicted molecular weight (Figure 1B). Only CD4/gp41^+ ^is shown in Figure 1B, the migrations of both CD4/gp41 proteins were similar and gave rise to the same major and a minor protein species. We found no evidence for proteolytic cleavage. The expression levels differed between the individual chimeric CD4 proteins. The two larger chimera containing the cytoplasmic portions of HIV-1 Env appeared to be expressed at slightly lower levels. A similar difference was observed by immunofluorescence of transfected cells (Figure 1C). Gentle permeabilization of the fixed cells allowed a comparison of intracellular and cell surface expression of the chimeric CD4 proteins. Each protein was transported to the plasma membrane and was available for potential incorporation into vector envelopes.

Insertion of the chimeric CD4 DNAs into the pCR3 plasmid allowed expression from either the CMV or T7 RNA polymerase promoter. After DNA transfection, the functionality of the CD4 proteins was initially evaluated in HeLa cells by a syncytia-forming assay. Infection of transfected HeLa cells with a vaccinia virus recombinant encoding T7 RNA polymerase allowed higher expression levels of the chimeric CD4 proteins in an increased number of cells. Co-infection of the cells with a vaccinia virus recombinant encoding the HIV-1 Env provided the cell fusion function. Syncytia formation was detected with all except the two CD4/gp41 chimeric proteins, however, as shown below, all CD4 chimera including the CD4/gp41 proteins were functional Env receptors after insertion into defective HIV-1 particles. Syncytia formation through CD4-Env interactions most likely requires a high density of CD4 expression at the cell surface and/or a conformational change, presumably neither of which were provided by the CD4/gp41 proteins.

### Defective HIV-1 packaging construct

**Figure 2:**
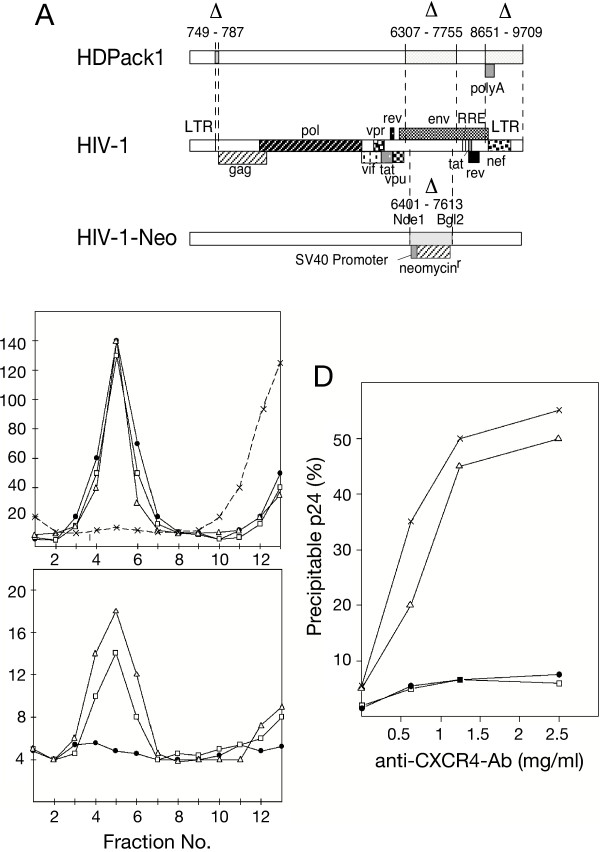
**Generation of HIV(CD4) particles. **(A) HDPack1. Three deletions were made in pNL4-3 DNA, an infectious clone of HIV-1 [62], and the SV40 polyadenylation site was added to create the defective HIV-1 helper virus construct, HDPack1. HIV-1-Neo is a defective HIV-1 DNA construct derived from HIV-1 HXB2 DNA with part of env replaced by the SV40 promoter and neomycin^r ^gene [63]. (B) and (C) Sucrose gradient fractionation of HIV(CD4) particles. HIV particles without CD4 or carrying either CD4 or CD4/gpi were isolated after transfection of HeLa, HeLaT4 and HeLaS2 cells with pHDPack1 DNA. The particles were concentrated by centrifugation onto a 65 % sucrose cushion followed by separation through a 15–60 % sucrose gradient. A portion of the particles isolated from HeLa cells was pretreated with Triton X-100 prior to centrifugation to solubilize the viral membrane. The p24 (B) and CD4 (C) concentrations of each gradient fraction were determined by ELISA. HIV(w/o CD4) particles (●), Triton X-100 treated HIV(w/o CD4) particles (X), HIV(CD4) (□) and HIV(CD4/gpi^+^) particles (△). (D) Immunoprecipitation of HIV(CD4) particles with anti-CXCR4 antibody. Pseudotype virions were isolated after co-transfections of HDPack1 and pCR3-CD4 DNA in either NIH 3T3 (□) and COS-7 (●) cells or human embryonic kidney 293 (△) and HeLa (X) cells. The amounts of immunoprecipitated p24 antigen were determined by ELISA and are shown as a percentage of the total amount of input p24 antigen.

A replication-incompetent HIV-1 packaging construct, HDPack1, was generated by deleting part of the HIV-1 packaging signal, most of Env and the entire Nef region of the infectious HIV-1 DNA clone pNL4-3 (Figure 2A). A SV40 polyadenylation site replaced the remaining part of the 3' LTR. Cotransfection of HDPack1 with HXN, a minimal vector construct that encodes a neomycin resistance gene [35], into BOSC cells that constitutively express the MMLV ecotropic Env protein [36], generated a MMLV Env^+ ^pseudotype lentiviral vector, which was able to confer neomycin resistance by stable transduction of NIH3T3 cells (data not shown). This demonstrated that HDPack1 provided all necessary structural proteins for pseudotype vector formation and for stable gene transfer.

### Incorporation of CD4 into vector envelopes

Cotransfection of HDPack1 with CD4 or any of the five chimeric CD4 DNAs into HeLa cells yielded approximately 15–25 ng p24/ml in the cell supernatant two days after transfection. Approximately one half of the total synthesized p24 antigen was released by the cells and could be immunoprecipitated by anti-CD4 antibody. Single DNA transfections of HDPack1 into HeLaT4 or HeLaS2 cells resulted in higher yields of CD4 carrying particles (over 100 ng/ml). HelaT4 and HeLaS2 cells constitutively express high levels of CD4 and CD4/gpi, respectively. Sucrose gradient analysis of supernatants from HDPack1 transfected cells revealed a peak of p24 antigen, which contained the vector particles (Figure 2B) harvested from HeLa, HeLaT4 or HeLaS2 cells. Over 90% of the p24 in cell supernatants were associated with either vector particles or microvesicles that are simultaneously released by the cell and cannot be separated by this gradient [37]. Solubilization of the viral membrane by 0.4% Triton X-100 shifted the p24 antigen to the top of the gradient (Figure 2B). The CD4 peak coincides with the p24 antigen peak (Figure 2C). 1 ng of p24 corresponds to 1–2 × 10^7 ^mature and immature virus particles. If all CD4 molecules in the cell supernatants were only associated with p24 containing vector particles and each contained 1000–2000 molecules of p24, we estimate that a single particle would carry approximately 30–130 CD4 or CD4/gpi molecules in their envelope. This estimate must be reduced by an unknown amount of CD4 or CD4/gpi molecules that could be associated with microvesicles.

### Insertion of CXCR4 into vector envelopes

During infection, HIV-1 uses CD4 as its primary receptor and a member of the chemokine receptor family like CXCR4 or CCR5 as a coreceptor [38,39]. It was uncertain whether endogenous levels of CXCR4 in HeLa cell membranes would be sufficient for incorporation. HIV-1 has been shown to exclude the insertion of the coreceptors CXCR4, CCR5 and CCR3 into its envelope [40]. Defective HIV-1 particles were generated by transfecting pHDPack1 DNA into either the human HeLa and Hek293 or mouse NIH3T3 and African green monkey COS-7 cell lines. Anti-CXCR4-antibodies precipitated fifty percent of p24 antigen of particles generated in the two human cell lines (Figure 2D), but not with particles generated in NIH3T3 or COS-7 cells. In a separate experiment, CD4^- ^HDPack1 particles isolated from HeLa cells could also be precipitated with anti-CXCR4 antibodies as efficiently as CD4^+ ^particles generated from HeLaT4 and HeLaS2 cells, suggesting that both, CXCR4 and CD4 are independently inserted into vector envelopes.

### Targeting and stable transduction of Env expressing cells

**Figure 3:**
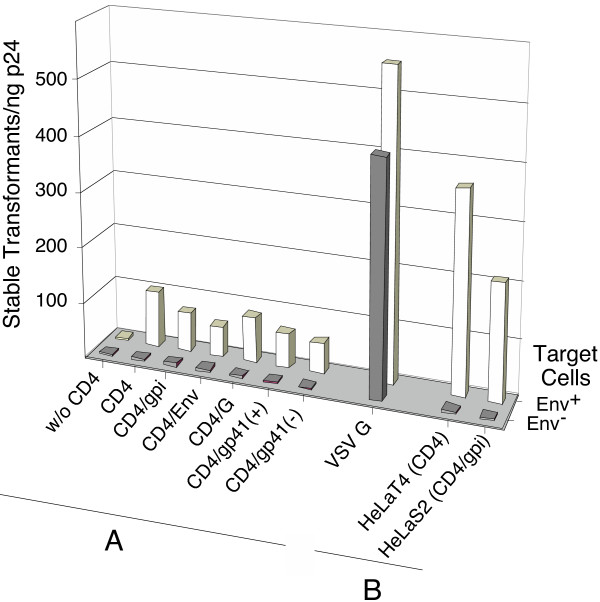
**Stable transduction of Env^+ ^cells by HIV-1-Neo(CD4/CXCR4) particles. **(A) Vector particles isolated from Hek293 cells after single transfections of HIV-1-Neo or after co-transfections of HIV-1-Neo with DNAs encoding either CD4 or the five individual chimeric CD4 proteins or VSV G protein. (B) Particles were harvested after single transfection of HIV-1-Neo into HeLaT4 or HeLaS2 cells stably expressing CD4 or CD4/gpi proteins, respectively. Equal amounts of particles (1 ng p24) were used to infect either 10^5 ^Env^+ ^TF228 or Env^- ^BJAB cells and neomycin-resistant cell colonies were selected.

Particles carrying the different chimeric CD4 proteins and CXCR4 were isolated from cell supernatants after cotransfections of Hek293 cells with chimeric CD4 and HIV-1-Neo DNAs. Equal amounts of these HIV-1-Neo particles (1 ng p24) were used to infect HIV-1 envelope protein expressing TF228 (Env^+^) cells as well as their parental BJAB (Env^-^) cells (Figure 3). The stable transduction of Env^+ ^cells demonstrated that the different chimeric CD4 proteins are functional receptors for Env and that the amount of endogenous CXCR4 expression in Hek293 cells is sufficient for functional incorporation into vector envelopes.

Transduction by the CD4/gp41 chimeras also demonstrates discordance between the ability of Env-CD4 interactions to mediate viral entry and syncytia formation, which was not observed with these two constructs. In comparison, HIV(VSV G) vector particles, generated by cotransfection of DNAs encoding the fusogenic VSV G protein and HIV-1-Neo, transduced both Env^+ ^and Env^- ^cells at similar high efficiencies of approximately 500 colonies/ng p24. This was about sevenfold more efficient than the average of 67 colonies/ng p24 by the different HIV-1-Neo(CD4) particles generated by DNA cotransfections. The differences in the expression levels of the various chimeric CD4 proteins as shown in Figure 2 were not reflected in the transduction efficiencies of the vectors.

In contrast, HIV-1-Neo(CD4) and HIV-1-Neo(CD4/gpi) particles generated by single HIV-1-Neo DNA transfections of HeLaT4 or HeLaS2 cells, respectively, had approximately three to fourfold higher transduction capabilities as compared to vector particles that were generated by the less efficient DNA cotransfections. This differs from HIV(VSV G) pseudotypes that were also generated by DNA cotransfections but have higher transduction efficiencies because, unlike CD4^+ ^particles, they are not dependent on the fusogenic activity provided by HIV-1 Env^+ ^expressing target cells.

### Kinetics of HIV(CD4) particle adhesion to Env^+ ^and Env^- ^cells

**Figure 4:**
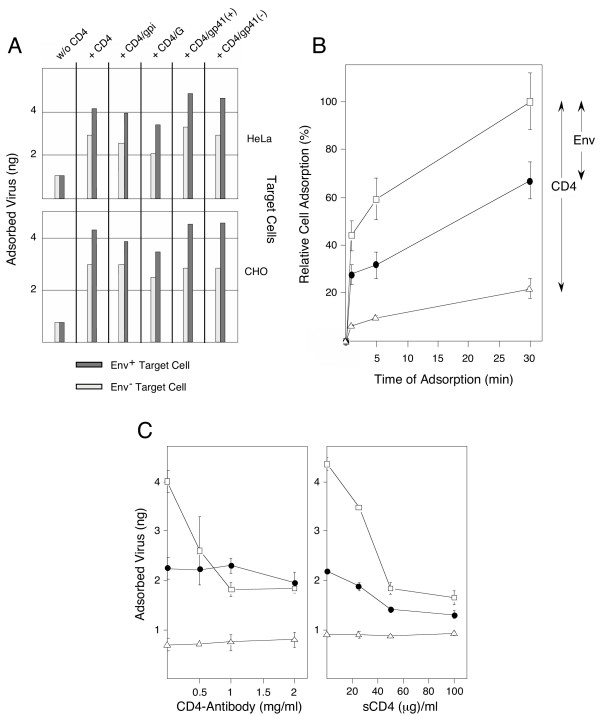
**Cell adhesion by vector particles. **Panel A: HIV particles without CD4, or carrying CD4 or one of the different chimeric CD4 proteins in their envelope, were generated by cotransfections with pHDPack1 in HeLa cells. Equal numbers (10 ng p24) of particles were adsorbed in suspension for 30 min at 4°C to 10^5 ^Env^+ ^or Env^- ^target cells (HeLa or CHO). The amount of adsorbed virus particles associated with the cell pellet was determined by p24 ELISA. Panel B: Time course of cell adhesion by HIV particles. Binding of HIV(CD4) particles to Env^+ ^cells (□), Env^- ^cells (●), or adhesion of HIV(w/o CD4) particles to Env^+ ^cells (△). Adsorption was normalized to the maximal amount of CD4^+ ^particles adsorbed to HIV Env^+ ^cells at 30 min (= 100). Contributions of the CD4-dependent, and the Env-dependent cell adhesion are indicated. Panel C: Inhibition of virus binding by either anti-CD4 antibody (left panel) or soluble CD4 (right panel). Equal p24 amounts of HIV(CD4) particles were adsorbed to either Env^+ ^(□) or Env^- ^(△) HeLa cells. The amounts of bound vectors were compared to the adsorption of HIV(w/o CD4) particles to Env^+ ^cells (△). Before cell adsorption, the vector particles were preincubated with increasing amounts of polyclonal antibody to CD4. Alternatively, with sCD4, the target cells were preincubated before addition of the vector particles.

Equal amounts of virus particles (ng p24) generated using HDPack1 in HeLa cells were added to HeLa and CHO cells that either transiently (HeLa cells infected with a vaccinia virus recombinant expressing Env) or stably (CHO-WT) express HIV-1 Env protein, respectively. Homologous Env^- ^cells (HeLa cells infected with vaccinia virus recombinant expressing T7 RNA polymerase or CHO-EE cells) served as a control. Unexpectedly, CD4^+ ^particles adsorbed to Env^+ ^and Env^- ^HeLa as well as CHO cells (Figure 4A). Though Env expression on target cells was absolutely required for transduction as shown in Figure 3, particles carrying CD4 adhered to Env^+ ^cells only about 1.5 times better than to Env^- ^HeLa or CHO cells. Adhesion to Env^- ^cells was significantly reduced when the particles did not carry CD4 in their envelope.

The kinetics of cell adhesion were similar for the five different HIV-1(CD4) particles. Positioning the CD4 ectodomain further outside the vector envelope, as in the CD4/gp41 chimeras, did not significantly diminish binding efficiency, suggesting that the observed cell adhesion is independent of the type of CD4 membrane anchor. A time course of vector binding at 4°C is shown in Figure 4B, which summarizes the contribution of the specific CD4-Env dependent interaction to the overall cell adhesion of the particles. In comparison, the particles adhered less well when they did not carry CD4.

The binding of soluble CD4 to Env can be enhanced by raising the temperature [41]. Particle adhesion was also compared at room temperature and at 37°C, which enhanced the binding activities proportionally. Significant differences in the relative Env-dependent and Env-independent cell adhesion by these particles were not detected at the three temperatures. Unexpectedly, the rate of Env-independent cell adhesion by CD4^+^particles appears comparable to the rate of the CD4-Env binding.

### Inhibition of cell adhesion by anti-CD4 antibodies and soluble CD4

The binding of CD4^+ ^particles to Env^+ ^cells was partially inhibited by addition of polyclonal anti-CD4 antibody (1 mg/ml). CD4^+^particles were pre-incubated with polyclonal anti-CD4 antibody before addition of Env^+ ^or Env^- ^HeLa cells (Figure 4C, left panel). Higher antibody concentration did not further reduce cell adhesion below the level observed for the binding of CD4^+ ^particles to Env^- ^cells. The low level of background binding seen with Env^- ^cells was unaffected, demonstrating that Env-independent cell adhesion was not blocked by polyclonal anti-CD4 antibody.

Preincubation of Env^+ ^and Env^- ^target cells with increasing concentrations of soluble CD4 specifically inhibited the binding of CD4^+ ^particles (Figure 4C, right panel). Similar concentrations of sCD4 (30 μg/ml) were needed to partially reduce Env-dependent and Env-independent cell adhesion. This suggests that the avidity of the Env-independent binding of CD4^+^particles to Env^- ^cells may be similar to that of the Env-dependent CD4 binding.

Although cell adhesion by CD4^+ ^particles (Figure 4C, left panel) was only partially inhibited by anti-CD4 antibody, transduction of Env^+^cells by HIV-1-Neo(CD4) and HIV-1-Neo(CD4/gpi) particles was completely blocked by anti-CD4 antibody as shown in Table 1. By comparison, HIV-1-Neo(G) pseudotypes, which carry the VSV G protein, were completely neutralized by anti-G antibody but not by high amounts of anti-CD4 antibody. Env^- ^cells were only infected by HIV-1-Neo(G) and not by HIV-1-Neo(CD4) particles. These results confirm the specificity of the antibodies used and demonstrate that the Env-independent cell adhesion by CD4^+ ^particles does not result in stable transduction. In contrast, the adhesion of particles without CD4 to Env^+ ^cells was low and unaffected by either anti-CD4 antibody or sCD4.

**Table 1:** Neutralization of vector infectivity by antibodies to CD4 and VSV G^a^

Antibody:	None^c^		anti-CD4^c^		anti-VSV G^c^	
Particle^b^	Env^+^	Env^-^	Env^+^	Env^-^	Env^+^	Env^-^
HIV-1-Neo(w/o CD4)	3	4	4	0	3	4
HIV-1-Neo(CD4)	403	5	6	3	401	3
HIV-1-Neo(CD4/gpi)	363	2	7	3	391	4
HIV-1-Neo(G)	591	517	446	550	4	6

^a^10^5 ^Env^+ ^TF228 or 10^5 ^Env^- ^BJAB cells were infected and neomycin-resistant cell colonies were selected in soft agar and counted after two weeks. The average number of selected colonies is presented from at least two titrations.

^b ^Vector particles were collected from human 293 cell supernatants after single DNA transfection with HIV-1-Neo or after cotransfection of HIV-1-Neo DNAs with DNAs encoding either CD4, CD4/gpi or the glycoprotein G of vesicular stomatitis virus, respectively.

^c^1 ng p24 of each vector type was preincubated for 30 min with or without 2 mg/ml antibody directed either against the CD4 ectodomain or the vesicular stomatitis virus glycoprotein G prior to infections.

### Vector generation in different host cells and targeting different cells

**Figure 5:**
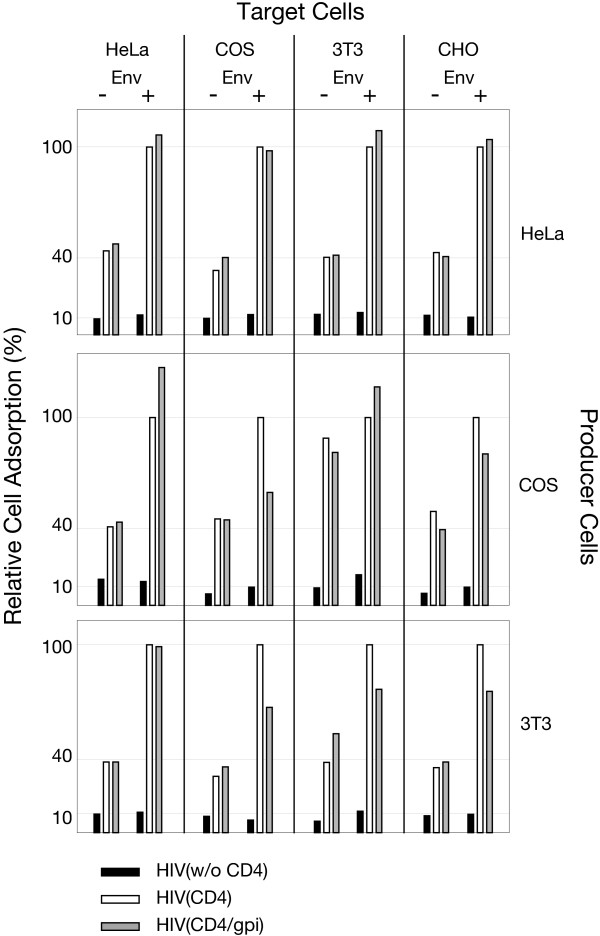
**Cell adhesion by CD4^+ ^and CD4/gpi^+ ^particles generated in different cell types. **Vector particles without CD4 or with CD4^+ ^or CD4/gpi^+ ^were generated in three different producer cell lines by DNA co-transfections of HDPack1 with CD4 or CD4/gpi encoding plasmids. Equal amounts of p24 vector particles (10 ng) were added to the same number of Env^+ ^or Env^- ^target cells (HeLa, COS-7, NIH 3T3 and CHO). Except for Env^+ ^CHO-WT and Env^- ^CHO-EE cells, the other Env^+ ^target cells were generated by high multiplicity infections with a vaccinia virus recombinant expressing either HIV-1 Env (+) or with T7 RNA polymerase (-) as a control. The amounts of adsorbed vector were normalized for each vector/target cell combination to the amount of HIV(CD4) vector adsorbed to Env^+ ^target cells (= 100).

To confirm that this newly observed cell adhesion property is unique to CD4 carrying vector particles, HIV(w/o CD4), HIV(CD4) and HIV(CD4/gpi) particles were produced from three different cell lines: human HeLa, African green monkey COS or mouse NIH3T3 cells. For cell adhesion, the same amounts of particles were added to Env^+ ^and Env^- ^HeLa, COS, CHO or NIH3T3 target cells. Cell adsorption of these particles is shown in Figure 5, which was normalized to the adsorbed CD4^+ ^vector of each vector/cell combination (= 100). Similar differences in cell adhesion were observed with CD4^+^, CD4/gpi^+ ^and particles without CD4 that were generated in the three cell types when added to the four different Env^- ^and Env^+ ^target cell types. The cell adhesion by CD4^+ ^and CD4/gpi^+ ^particles from all cell types suggests that the presence of the CD4 ectodomain plays an important role in Env-independent cell adhesion by these particles.

### Diminished targeting of Env^+ ^cells in Env^+^/Env^- ^cell mixtures

Expression of a chimeric CD4/Env protein from the HIV-1 LTR has previously been shown to decrease HIV-1 spread in vitro, however, stable targeted transduction of HIV-1 Env expressing cells by HIV(CD4) particles was not detected by us previously [6,7]. A lack of CXCR4 insertion can now be ruled out by the data presented above. We hypothesized at the time that even at a low amount of Env^+ ^cells in the target cell population resulting from less efficient transfections with Env-encoding DNA, the highly specific cell targeting should remain effective unless access to Env^+ ^target cells was simply blocked physically by a high cell density of Env^- ^cells. The avid, Env-independent cell adhesion by CD4^+ ^particles described above, however, could potentially provide an alternative explanation.

**Figure 6:**
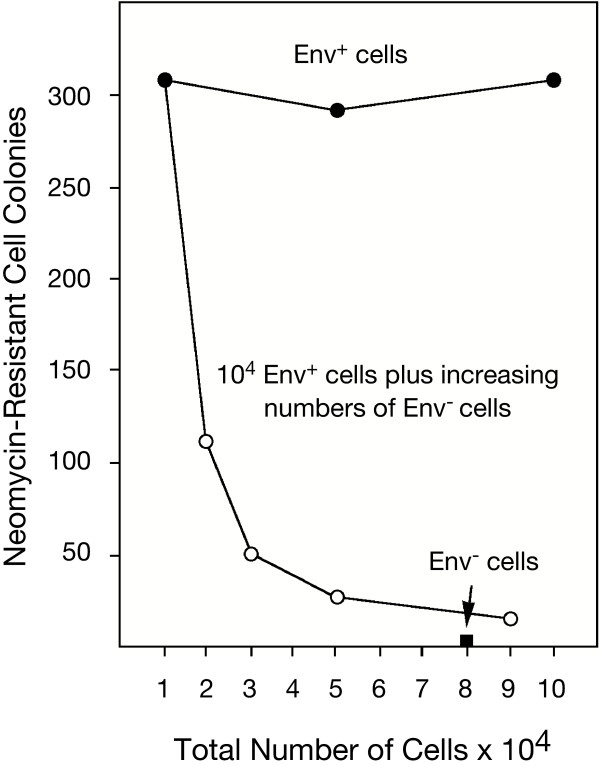
**Diversion of targeted HIV-1-Neo(CD4/CXCR4) particles. **One ng of HIV-1-Neo(CD4/CXCR4) particles was adsorbed to homogenous populations of either Env^+ ^TF228 (•) or Env^- ^BJAB cells (■), or to mixed cell populations consisting of 10^4 ^Env^+ ^cells plus increasing amounts 10^4^-8 × 10^4 ^of Env^- ^cells (○). Stably transduced cells were selected in the presence of neomycin.

To determine the particle to cell ratio at which transduction is saturated, a constant amount of HIV-1-Neo(CD4/CXCR4) particles (1 ng p24) was added to a cell suspension consisting of increasing amounts (10^4 ^to 10^5^) of Env^+ ^TF228 cells. The total volume of the suspension was maintained at 0.5 ml DMEM with 10% serum. Virus particles were adsorbed for 40 min at 37°C. To prevent cell sedimentation and to promote free access and random interactions of the cells with the vector, the suspensions were mixed every 10 min. Neomycin-resistant transformant colonies were subsequently selected for two weeks in agar and counted. Independent of the total number of Env^+ ^target cells present, a constant number of approximately 300 neomycin-resistant colonies was selected. This indicated that the amount of vector particles in the cell suspension limited the total number of stable transformants within the density range of Env^+ ^target cells tested (Figure 6).

To evaluate the efficiency of Env^+ ^cell targeting, Env^+ ^(TF228) and Env^- ^(BJAB) cells were mixed at defined ratios. A constant amount of HIV-1-Neo(CD4/CXCR4) particles was added to cell suspensions with increasing ratios of Env^- ^to Env^+ ^cells. Each population contained 10^4 ^Env^+ ^TF228 cells and increasing numbers (10^4 ^to 8 × 10^4^) of Env^- ^BJAB cells, the parental cells of TF228. As shown in Figure 6, addition of increasing numbers of Env^- ^cells dramatically decreased the number of neomycin resistant colonies. At a 1:1 ratio of Env^+^:Env^- ^cells and a total number of 2 × 10^4 ^cells, the number of transformants was reduced over fifty percent. While transduction of Env^+ ^cells in this mixed cell population remained selective, the efficiency of cell targeting was severely diminished. The low cell density in this relatively large volume of medium, together with the repeated mixing of the cell suspension make it unlikely that Env^- ^cells physically blocked access of the vector to Env^+ ^target cells.

These results suggest that the Env^- ^cells effectively competed with Env^+ ^cells for the binding of HIV-1-Neo(CD4/CXCR4) particles. If this competition was caused by CD4-dependent adhesion to Env^- ^cells as demonstrated in Figures 4 and 5, we would infer that its rate of cell binding would be similar to that of the specific binding of HIV-1-Neo(CD4/CXCR4) particles to Env^+ ^cells. Consequently, stable transduction of Env^+ ^cells may be severely diminished by this highly effective CD4-dependent adhesion to Env^- ^cells.

## Discussion

Accomplishing functional insertion of CD4 and CXCR4 into HIV-1 vector envelopes is the initial step towards targeting HIV-1 infected cells with the future long-term goal to inhibit HIV-1 replication. The assembly and functionality of vector particles carrying different chimeric CD4 proteins in their envelopes revealed that protein insertion into vector envelopes was readily achieved by either DNA co-transfections or by using cells that constitutively express CD4 or CD4/gpi. Significant differences in the targeted transduction of Env^+ ^cells were not detected after replacing the transmembrane and cytoplasmic domains of CD4 with corresponding regions of either the HIV-1 envelope protein, the VSV glycoprotein G or the gpi anchor of the cellular DAF protein [42]. This indicates that the origin of the CD4 anchor was not critical for receptor function during infections, which is consistent with earlier observations that Env binding needed for infection maps near the amino terminus (amino acids 40–60) of CD4 [43].

For targeted transduction of cells that constitutively express the T cell tropic HIV-1 Env protein, vector insertion of both, CD4 and the CXCR4 coreceptor is needed. The level of endogenous CXCR4 expression in HeLa cells was sufficient for functional insertion of the coreceptor into vector particles and CXCR4 DNA co-transfection was not required. We conclude that HIV-1 readily inserts many viral and nonviral membrane proteins, and a large number of cellular proteins have previously been identified in HIV-1 envelopes [11,14].

The transduction efficiencies of six different HIV(CD4) pseudotypes generated by co-transfection of HIV-1-Neo with a CD4 expression plasmid were very similar, giving rise to approximately 70 neomycin-resistant colonies per ng p24 (Figure 3). HIV-1-Neo DNA transfection of HeLaT4 or S2 cells, which stably express CD4 and CD4/gpi, respectively, produced higher titers of pseudotyped particles yielding transduction efficiencies of approximately 280 colonies per ng p24. This compares favorably with wild-type HIV-1, which, at 1–3 × 10^3 ^infectious units per ng p24 [44], is only three to ten fold more infectious. Thus exchanging the roles of viral and cellular membranes during membrane fusion does not dramatically reduce viral infectivity.

HIV-1-Neo(CD4/CXCR4) particles, independent of the type of CD4 anchor, were able to target and selectively infect cells expressing T cell tropic HIV-1 Env. In homogenous Env^+ ^cell populations, this transduction was efficient. Unexpectedly, CD4^+ ^particles also adhered very efficiently to a variety of human and animal cell types that do not express HIV-1 Env without leading to cell transduction. The competition between Env^+ ^and Env^- ^cells for CD4^+ ^particles in mixed cell populations (Figures 5 and 6), the time course of CD4^+ ^particle binding to Env^+ ^and Env^- ^cells (Figure 4B) and the inhibition of CD4^+ ^particle adhesion by anti-CD4 antibody and by sCD4 (Figure 4C) all suggest that the specific Env-dependent and the Env-independent cell binding by these particles may have similar avidity. CD4-Env binding has a dissociation constant (K_D_) of 5 nM, which is comparable to that of a good antibody-antigen binding complex [45]. Our studies focused on the adhesion of entire virus particles to cells. Polyclonal anti-CD4 antibodies, which blocked HIV-1-Neo(CD4/CXCR4) or HIV-1-Neo(CD4/gpi/CXCR4) transduction of Env^+ ^cells (Table 1), did not completely prevent Env-independent cell adhesion (Figure 4C, left panel). This suggests either a CD4 domain that may be involved in the Env-independent adhesion was not blocked by the antibody, or alternatively, CD4 may have recruited an additional membrane component into the vector envelope, which causes the new cell adhesion by the particles. In the latter case, the same or a similar membrane component must be present on the various cell types used in the studies.

The efficiency of this Env-independent cell adhesion by CD4^+ ^particles was unexpectedly high. At a ratio of 1 Env^+ ^to 8 Env^- ^cells, the transduction of Env^+ ^cells was inhibited by over ninety percent (Figure 6). HIV-1 is a cytolytic retrovirus and the number of cells expressing HIV-1 Env *in vivo *are few [46-48]. Although many silently infected cells are present in lymph nodes, only a small fraction (1 in 300) generally expresses HIV-1 Env [49]. Since Env-independent cell adhesion occurred with all CD4^+ ^particles and all cell types tested (Figure 4A and 5), we predict that targeted HIV(CD4) particles will be unable to infect Env^+ ^cells at high efficiency *in vivo*. This severely limits, if not totally abolishes, the feasibility of such an antiviral approach. Transduction of Env^+ ^cells by HIV(CD4/CXCR4) particles, similar to the particles used in the present study, has previously been reported [11], however the efficacy of cell targeting was not evaluated in mixed cell populations. Without this challenge, the potential limitations for cell targeting *in vivo *were not recognized. Earlier assumptions that HIV(CD4/CXCR4) particles could potentially target HIV-1 infected cells in patients relied on the high specificity and presumed strength of the CD4-Env interaction.

The first clinical trial using a HIV-1-based vector is currently in progress [50,51]. A vector was introduced at high efficiency into patients T4 lymphocytes *ex vivo*. The purpose of this trial is to evaluate the safety of the HIV-1-based vector and to render the cells resistant to endogenous HIV-1 strains, which previously did not respond to antiviral drug cocktails. For gene therapy, effective transgene expression *in vivo *generally requires, besides very high *in vivo *grade vector titers, optimal access to the target cell population. The *ex vivo *transduction of enriched human T-lymphocytes by a HIV-1-based vector can be expected to be much more efficient because this route of transgene delivery avoids vector infusion into complex cell populations or target tissues.

Vector distribution *in vivo *is likely to be affected by proteins that are inserted into the vector envelope during viral budding from the producer cell. The unexpected diversion of targeted HIV(CD4/CXCR4) particles has important general implications for the use of enveloped viral vectors *in vivo*. It demonstrates that the vector producer cell can greatly impact on the transduction efficiency of the vector depending on the nature of the target cell within a complex cellular environment *in vivo*. Insight from detailed studies by several labs [19-22,52,53] can be expected to help with the choice of vector producer cells for specific cell targeting applications in the future.

Our study compared the cell adhesion and the infectivity of vectors produced in the absence of Vpu, Nef and Env expression, which allowed to focus on the immediate effects of CD4 and CXCR4 insertion into vector envelopes. During wild-type HIV-1 infection, CD4 and the coreceptors CXCR4 and CCR5 are excluded from the HIV-1 envelope through interactions with HIV-1 Nef protein [40,54]. The insertion of endogenous CXCR4 from HeLa and Hek293 cells was sufficient for the generation of infectious HIV-1-Neo(CD4/CXCR4) particles. Without Vpu, Env and Nef expression from both HDPack1 and HIV-1-Neo, insertion of CD4 and CXCR4 appeared undisturbed and the resulting vector particles efficiently infected homogeneous Env^+ ^target cell populations. In fact, their infectivity was surprisingly effective and not dramatically reduced as one might anticipate in comparison to particles carrying the fusogenic VSV glycoprotein.

CD4 down-modulation benefits the release and the infectivity of the released particles. Whether CD4-dependent membrane adhesion already occurs intracellularly in the absence of HIV Env is currently unknown. As shown here, functional vector particles were efficiently released. Additional expression of Env interferes with CD4 cell surface expression and vice versa. Particles containing both Env and small amounts of CD4 could potentially be released from the cell. Upon release, these particles may either adhere directly to the cell from which they originated, or they may bind to and be arrested by adjacent non-host cells. In fact, a chimeric CD4 protein, which lacks the C-terminus and is not down-modulated by proteolytic degradation [55,56] may effectively inhibit HIV-1 replication as previously suggested [7].

Vpu plays an important role in the down-modulation of CD4. Vpu forms cationic-selective channels [57]. Amiloride derivatives have been shown to be able to block Vpu channel activity and to reduce HIV-like particle release and viral replication in human macrophages [58,59]. From our studies presented here, we infer that the reduced amount of particles released form T4 cells and macrophages in the absence of functional Vpu (or Nef) might carry increased amounts of CD4 in their envelope and thereby potentially decrease the HIV-1 infectivity through cell adhesion to a majority of non-host cell types. More detailed studies on the precise mechanism of the cell adhesion and the resulting particle diversion are necessary to better understand the potential involvement of CD4. If confirmed, inhibiting Vpu and/or Nef activity may potentially help augment the effectiveness of the current highly active antiretroviral therapy.

## Conclusion

Lentiviral vectors targeted to HIV-1 expressing cells could potentially be part of an antiviral strategy. HIV-1-based vectors, which carry CD4 in their envelope, do selectively infect HIV-1 Env expressing cells, however, an unexpectedly avid and Env-independent cell adhesion by the same CD4^+ ^particles diminished the targeted transduction of Env^+ ^cells in mixed cell populations. Further studies on the potential direct or indirect role of CD4 during this cell adhesion are needed, which may provide an additional rationale for the multiple efforts by HIV-1 to down-modulate CD4 expression prior to viral assembly. Without CD4 down-modulation, insertion of CD4 into virus particle envelopes may reduce infectivity, thus viral mechanisms to down-modulate CD4 could potentially become antiviral targets. The apparent ease of inserting cellular proteins from vector producer cells into HIV-1 envelopes has important implications for transgene delivery by HIV-1-based vectors as well as other enveloped viral vectors. Depending on the target cell and its cellular environment *in vivo*, the choice of vector producer cells could affect infectivity and thereby vector efficacy during gene therapy.

## Materials and methods

### Cell types and cell culture

HeLa, HeLaT4 expressing CD4 protein [1], HeLaS2 expressing CD4/gpi protein (Ragheb JA: unpublished), COS-7, Hek293 cells and NIH3T3 cells were grown in Dulbecco's minimal essential medium (DMEM; GIBCO), 10% fetal bovine serum (FBS), 1% penicillin/streptomycin. Chinese hamster ovary cells with (CHO-WT) or without (CHO-EE) HIV-1 Env expression were grown in GMEM-S media [60]. The human lymphoblastoid cell line BJAB and its HIV-1 Env expressing progeny TF228 [61] were maintained in DMEM/16 % FBS/pen/strep suspension cultures. Neomycin resistant colonies were selected with 400 μg neomycin/ml in soft agar.

### Chimeric CD4 proteins

(A) pCR3-CD4/gp41: CD4 and gp41 protein coding regions derived from pHD1 [7] and pNL4-3 [62] were fused by PCR [5] at pos. 1250 in CD4 and at pos. 7705 in pNL4-3 using the fusion primer CCCCGGTGCAGCCAATGATTGAACCATTAGGAGTAGC and the terminal primers AAGCTTGGTTACCCAGGACC and GGAGTGTATTAAGCTTGTG. The fusion product was ligated at HindIII to gp41, pos. 8145 to 8887 of pNL4-3. A 1680 bp BstEII/BssHII fragment of CD4/gp41 was transferred into pCD4/G [5]. The complete CD4/gp41^+ ^coding region (Xba1-BssHII) was blunt-end ligated at EcoRV into pCR3 downstream of the CMV promoter. With CD4/gp41^-^, the proteolytic cleavage site Ile-Glu (ATT-GAA) was deleted in the primers. (B) pCR3-CD4/G:CD4/G was excised from pCD4/G using XhoI and BssHII. After filling in with Klenow fragment, blunt ended CD4/G was cloned into pCR3 at EcoRV. (C) pCR3-CD4/Env: A 1085 bp fragment (BstEII-XhoI) was removed from pHD1 [7] and cloned into BstEII-XhoI of pCR3-CD4/G. (D) pCR3-CD4: Part of the CD4 coding region (603 BstXI-1737 BamHI) was removed from pT4B [1] and cloned at BstX1 and BamH1 of pCD4/G. (E) pCR3-CD4/gpi was constructed by inserting a EcoRI-HpaI fragment from the CD4/gpi [34] present in LA4SN (Ragheb JA: unpublished) into EcoRI-EcoRV of pCR3. Chimeric CD4 proteins were analyzed by Western blot using monoclonal anti-human CD4 (Leu-3a) with either [^125^I]-labeled S. aureus protein A or [^125^I]-labeled sheep anti-mouse whole IgG antibody and visualized by autoradiography. For immunofluorescent staining, cells were fixed with 3% formaldehyde with and without permeabilization by 0.4% Triton X-100. Primary mouse monoclonal antibody or polyclonal sheep anti-human CD4 protein serum were used in combination with secondary donkey anti-mouse IgG or anti-sheep IgG, conjugated with fluorescent isothiocyanate (FITC).

### Assembly of pHDPack1

For the generation of the HIV-1 packaging construct, several deletions were introduced into pNL4-3 (1) DNA by PCR. (A): a 654 bp fragment was amplified using primer (+) GAAGCGCGCACGGCAAGAGGCGAGGGGCGGCGACTGGTGAGAGATGGGTGCGAGAGCGTCGG and primer (-) GGCCCTGCATGCACTGGATG, deleting 39 bp (749–787), and cleaved with BssHII and SphI. pNL4-3 was cleaved with SphI (pos. 1404) and EcoRI (pos. 5743) resulting in a 4.3 kb fragment. Both fragments were ligated into BssHII and EcoRI of pHD1 [7]. (B): PCR of pNL4-3 with the terminal primers (+) CATAATAAGAATTCTGCAAC and (-) CAAGTTAACAGCACTATTC and the fusion primers (+) GGGATATTGATGTCTGTAGAATAGGAGCTTTGTTCCTTGGG and (-) CCCAAGGAACAAAGCTCCTATTCTACAGTCATCAATATCCC produced a 1457 bp fragment with a 1448 bp deletion in Env (pos. 6307–7755). It was cleaved with EcoRI and HpaI. (C): A 240 bp fragment, containing the poly(A) site of SV40, was amplified using (+) TAGCCCGGGATAAGATACATTGATGAGT and (-) TAGGAATTCATCATAATCAGCCATACCAC and cleaved with SmaI and EcoRI. The DNA from step (A) was cleaved with EcoRI and the fragments from steps (B) and (C) were inserted in a three-piece-ligation to generate pHDPack1. The defective HIV-1-Neo construct has previously been described [63]. Both, HDPack1 and HIV-1-Neo lack complete Vpu, Nef and Env coding regions.

### Generation of defective HIV-1 particles

HeLa, HeLaT4, HeLaS2, COS-7, 293 and NIH3T3 cells, grown in 35 mm dishes were cotransfected with 2.5 μg of pHDPack1 DNA and 2.5 μg of either CD4 or chimeric CD4 DNA using calcium phosphate coprecipitation. 48 hrs after transfection, the medium was centrifuged and the supernatant was filtered through 0.45 μm filter. The p24 concentration was determined and the virus was frozen at -70°C. With CD4^+ ^HeLaT4 and CD4/gpi^+ ^HeLaS2 cells, particles were generated after single transfections of either HDPack1 or, when stable transductions were evaluated, with HIV-1-Neo DNA. As a control for the functionality of HDPack1 as a helper construct that provides all necessary structural proteins, HXN(MMLV) pseudotypes were generated by cotransfection of HDPack1 and HXN DNAs [35] into BOSC cells, which express ecotropic Moloney murine leukemia virus Env protein [36]. With select particles, vesicular stomatitis virus glycoprotein G (Indiana serotype) was inserted by co-transfection.

For separation on sucrose gradients, the particles were concentrated by centrifugation onto a 65% sucrose pad at 150,000 × g for 60 min, resuspended in 0.5 ml of PBS and separated on 4.5 ml continuous 15–60% sucrose gradients in PBS at 120,000 × g for 2 hours. P24 and CD4 of each fraction were determined by ELISA. CD4^+ ^particles were precipitated with approximately 0.5 mg/ml of polyclonal antibody to human CD4 or rabbit anti-CXCR4 antibody (Millennium Biotechnology, CA). Antigen/antibody complexes were pelleted after binding to Protein-A-Sepharose. The amounts of p24 antigen present in solubilized virus particles were determined by ELISA. Polyclonal rabbit anti-VSV G protein antibody was used to neutralize HIV(G) pseudotype particles.

### Enzyme-linked immunoadsorbent assay for p24 and CD4 proteins

HIV-1 p24 protein was determined by p24 ELISA (Cellular Products Inc.). CD4 was quantified by a modified ELISA using 96 well Immulon-4 plate (Dynatech Laboratories, Inc.) next to CD4 protein standards (Intracel Corporation) and 50 μl of anti-CD4 polyclonal antibodies (5 μg/ml), followed by addition of 0.15 μg of alkaline phosphatase-conjugated anti-sheep IgG (Jackson ImmunoResearch Laboratories, Inc.) and nitrophenylphosphate as a substrate. The reaction was stopped by 0.1 M EDTA and quantified at 405 nm.

### Viral cell adhesion assay

Target cells were grown to 85% confluence. HeLa, COS-7 and NIH3T3 cells were infected at a multiplicity of infection of 5 with a vaccinia virus expressing either HIV-1 Env (vPE16) [64,65] or T7 polymerase (vTF7) [66]. Recombinant vaccinia virus infected cells as well as noninfected stable CHO-WT Env^+ ^cells and control CHO-EE cells were incubated at 37°C for 24 hours. The cells were suspended using a cell scraper and passed through a #16 needle. Cells were washed with PBS and resuspended in DMEM/10% FBS before virus adsorption. BJAB and TF228(Env^+^) suspension cells were washed before adding virus.

Each cell adhesion reaction contained 10^5 ^cells and 10–20 ng p24/ml viral particles in a total volume of 0.5 ml. Each reaction was incubated for 30 min at 4°C in 0.5 ml. After adsorption, the cells were washed, pelleted in 10% FBS medium and resuspended in 200 μl DMEM. P24 antigen of solubilized particles was determined by ELISA.

## Competing interests

The author(s) declare that they have no competing interests.

## Authors' contributions

Both, ZY and GGH carried out DNA clonings, vector isolations and p24 assays. ZY helped design cell binding assays and stable cell transductions. JAR generated the CD4/gpi chimera and CD4/gpi expressing HeLaS2 line, contributed to discussions and the draft of the manuscript. MS conceived of the study, designed most DNA constructs and experiments and drafted the manuscript.
